# Heavy and light roles: myosin in the morphogenesis of the heart

**DOI:** 10.1007/s00018-012-1131-1

**Published:** 2012-09-06

**Authors:** Jennifer England, Siobhan Loughna

**Affiliations:** School of Biomedical Sciences, University of Nottingham, Queens Medical Centre, Derby Road, Nottingham, NG7 2UH UK

**Keywords:** Myosin, Myosin heavy chain, Myosin light chain, Heart, Sarcomere, Development, Congenital heart defects

## Abstract

Myosin is an essential component of cardiac muscle, from the onset of cardiogenesis through to the adult heart. Although traditionally known for its role in energy transduction and force development, recent studies suggest that both myosin heavy-chain and myosin light-chain proteins are required for a correctly formed heart. Myosins are structural proteins that are not only expressed from early stages of heart development, but when mutated in humans they may give rise to congenital heart defects. This review will discuss the roles of myosin, specifically with regards to the developing heart. The expression of each myosin protein will be described, and the effects that altering expression has on the heart in embryogenesis in different animal models will be discussed. The human molecular genetics of the myosins will also be reviewed.

## Introduction

Congenital heart defects (CHDs) refer to anomalies in the structure of the heart or great vessels that are present at birth, and occur with a frequency of approximately 0.8 % (one in 145 live births; British Heart Foundation http://www.bhf.org.uk). As CHDs account for nearly one-third of all major congenital defects, they are the most common defect in newborns [1]. The heart forms early in development, with a linear cardiac tube present in the midline of the embryo at day 22 in humans. This tube undergoes rapid morphological changes to give rise to a correctly aligned and septated four-chambered structure by the end of the seventh week of human embryogenesis. Several structural proteins that are expressed in the heart are now known to be essential for cardiogenesis. A number of the genes to these structural proteins give rise to CHDs upon mutation, with mutations in myosin heavy chain 6 the first to be associated in 2005 [2]. The myosin II hexameric molecule is composed of one pair of heavy chains and two pairs of light chains. Myosin heavy chain (MHC) proteins can be broadly classified into two groups; the sarcomeric (cardiac and skeletal) and nonsarcomeric (smooth muscle and nonmuscle) myosins. The myosin light-chain (MLC) proteins are also classed into two groups, the essential and regulatory light chains. This review will provide an overview of the roles the myosin proteins play in the developing heart and their potential to give rise to CHDs. The known molecular genetics in humans and animal models will be discussed.

## Myofibrillogenesis in the developing heart

**Fig. 1:**
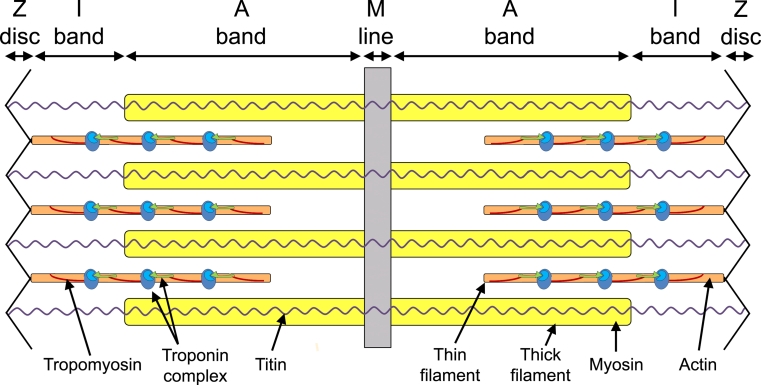
Schematic representation of a sarcomere. The thick and thin filaments overlap in the region of the A-band, with the I-band formed from the thin filaments only. The central M-line anchors the thick filaments and the Z-disk the thin filaments. Titin is found along the length of the sarcomere. Tropomyosin and the troponin complex interact with actin to form part of the thin filament

The heart is the first functional organ to develop in the vertebrate embryo due to the formation of myofibrils in cardiomyocytes that allow for muscle contraction. The sarcomere is the basic contractile unit of striated muscle, which is made of thick and thin filaments responsible for the generation of coordinated contractions. These sarcomeres unite to form individual myofibrils that align along the longitudinal axis of the rod-shaped cardiomyocytes. Myofibrils are highly ordered structures brought together by three components: actin and myosin filaments, accessory proteins of actin and myosin, and scaffolding proteins. A sarcomere spans between two Z-discs to which the thin filaments (actin-based) anchor and form the I-bands (Fig. 1). Myosin, the major component of the thick filament, is interdigitated between the actin-containing thin filaments creating A-bands in the center of the sarcomere. Thick filaments are held in place by an M-line, the central most structure of the sarcomere (Fig. 1). Titin is anchored to the Z-discs at its N-terminus, and the M-line at its C-terminus, and is thought to be important for the assembly of the sarcomeric proteins [3] (Fig. 1). In cardiac muscle, myofibrils from individual cells are joined by intercalated discs, structures that are analogous to Z-discs that contain terminal ends of actin filaments of the sarcomere, but also act as an adherens junction between cardiomyocytes. These intercalated discs ensure mechanical coupling within the working myocardium [4, 5]. Any impairment within the sarcomere can lead to dysfunction of the cardiac cells, and is therefore potentially detrimental to heart formation and function and hence to the embryo as it develops.

Myofibrillogenesis has become one of the most studied phenomena in development since structural proteins of the cardiac sarcomere were linked to myopathies (both cardiac and skeletal) and CHDs. Investigations into myofibrillogenesis have utilized the chicken heart as an animal model of the human heart. Contractions of the chicken heart initiate in the nine somite (HH10 or 36 h in ovo) embryo and just a few hours later, the cardiovascular system is so far developed that cardiomyocytes can pump blood throughout the embryo [6]. In fact, major components of the sarcomere are already expressed at the 6-somite (HH8) stage embryo prior to heart formation, where two regions of cardiogenic mesoderm containing premyocardial cells exist [7]. Therefore, myofibril assembly is an extremely rapid process that occurs early in development.

**Fig. 2:**
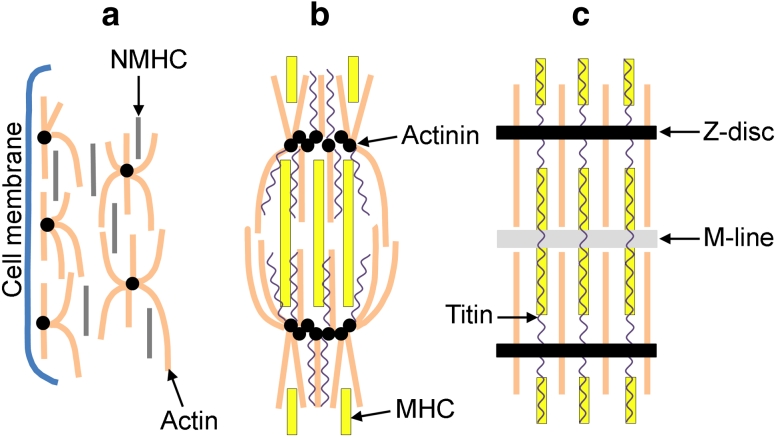
The assembly of myofibrils. **a** Formation of premyofibrils. Proteins assemble into structures known as premyofibrils, which characteristically contain α-actinin along the periphery of the cell, and nonmuscle myosin (NMHC) scattered between the actin. **b** Formation of nascent myofibrils. Muscle-specific myosin II isoforms and stabilizing proteins become incorporated into the myofibrils, with the myosin heavy-chain (MHC) proteins replacing NMHC. Titin is also expressed in the myofibril at this stage. **c** Formation of mature myofibrils. Nascent myofibrils fuse to form mature myofibrils, forming a highly organized sarcomeric structure

Myofibrillogenesis is a process containing a number of key steps including the formation of premyofibrils, nascent myofibrils, and mature myofibrils [8, 9]. Firstly, proteins assemble into structures known as premyofibrils, which resemble mini-sarcomeres (Fig. 2a). The first marker for the assembly of premyobrils is Z-bodies containing α-actinin along the periphery of the cell [9]. Actin monomers are incorporated between these Z-bodies forming actin filaments until the myofibrils reach their mature stage [10]. In addition, at the stage of the premyofibril, nonmuscle myosin IIB is located distinctly between the α-actinin containing Z-bodies (5-somite stage). Muscle myosin II is also present in these cells, and can even be detected before myofibrils begin to assemble (as early as the 3-somite stage), as rodlets of 0.76 μm. However, the muscle myosin remains scattered around the nucleus of the cardiomyocytes [8]. Secondly, the premyofibrils develop into nascent myofibrils that incorporate muscle-specific myosin II isoforms and stabilizing proteins (Fig. 2b). As nascent myofibrils develop, muscle myosin II begins to replace the nonmuscle isoform, and becomes distributed throughout the myofibril (~25 μm in length). Titin is also expressed in the myofibril at this stage and is inserted into the Z-discs, thus playing its role in maintaining and organizing the structures of the sarcomere, including myosin integration [11, 12]. Finally, nascent myofibrils begin to fuse to one another to form mature myofibrils, which contain a highly organized structure of A-bands and Z-discs, composed of structural sarcomeric proteins (Fig. 2c) [8]. By the 9-somite stage (HH10), when the first cardiac contractions have occurred, muscle myosin II has replaced the nonmuscle myosin and appears as highly organized bi-polar filaments of 1.6 μm in length [8, 13].

## Structure of the myosin molecule

**Fig. 3:**
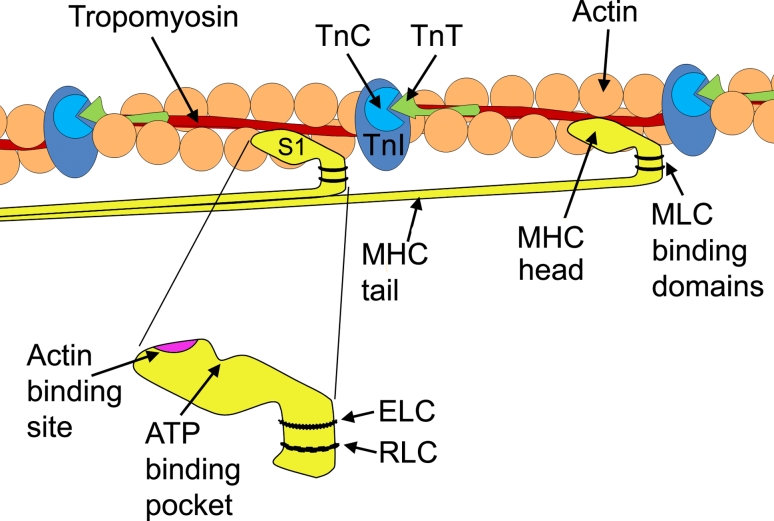
Simplified view of the relationship between the globular head of myosin heavy chain and the thin filament. The positions of tropomyosin and TnC, TnI, and TnT along the actin filament are illustrated. A magnification of the globular head shows the position of the actin-binding site, the ATP pocket, and the essential light chain (ELC) and regulatory light chain (RLC) binding domains. *MHC* myosin heavy chain, *MLC* myosin light chain, *S1* subfragment-1, *Tn* troponin

Myosin is a large, ubiquitous, motor protein that generates force through its interaction with actin, thus involving it in a number of cellular processes including cytokinesis, karyokinesis, cell migration, and muscle contraction [14]. Myosins can be divided into two distinct classes, the conventional two-headed myosins and the unconventional single-headed ones [15]. For the purpose of this review, we will be discussing the conventional class, which can be further subdivided into sarcomeric and nonsarcomeric myosins. The two-headed myosins are hexameric proteins (520 kDa) [14] comprising two myosin heavy-chain subunits and four myosin light-chain subunits (two regulatory and two essential light chains) [16]. The MHCs fold together at their C-terminus, thus forming a dimerized coiled-coil α-helix known as the tail region. This region contains the binding sites for myosin assembly into the sarcomere (e.g., titin and myosin-binding protein-C), and functions to anchor and position the motor domains of myosin so that it interacts with actin [17]. At the N-terminus, each MHC folds onto itself forming a globular head region (subfragment-1), so that each myosin molecule contains two globular heads. These head regions contain the binding sites for the MLCs, ATP, actin and divalent cations, and are called the motor region of the molecule (Fig. 3) [14].

The subfragment-1 domain of a myosin molecule is asymmetrical and contains a MHC folded with two MLCs. The subfragment-1 domain can be further subdivided into two regions; the motor domain and the regulatory domain, which links the motor domain to the tail of the myosin molecule [18]. Proteolysis of the myosin head reveals three major segments; a 25-kDa N-terminal portion, a central 50-kDa segment which together form the motor domain, and a 20-kDa C-terminal region, containing the regulatory domain [19]. The 50-kDa segment is separated into an upper and lower subdomain by a long, narrow cleft containing an actin-binding site. The 25-kDa segment attaches to the 50-kDa domain with an ATP-binding pocket located at the site of attachment. The 20-kDa segment is the site to which one regulatory and one essential light chain bind. It is the most extended region, formed by a long α-helix [18]. This region is thought to amplify small conformational changes into large movements needed to produce force, and therefore, sarcomeric contraction [20].

The MLCs are comprised of two sub-families, the essential or alkali MLC (MLC1 or ELC) and regulatory MLC (MLC2 or RLC), which have molecular masses of 22 and 19 kDa, respectively. The MLCs wrap around the α-helical neck region (20-kDa region) of the MHC in an anti-parallel orientation and stabilize it. The arrangement of MLCs relative to the ATP-binding site and actin-binding sites of the motor domain suggests a function in creating a longer molecule to amplify power stroke [18]. MLCs belong to the EF-hand family, a large family of Ca^2+^-binding proteins also associated with calmodulin and troponin-C [21]. MLCs contain two Ca^2+^-binding EF-hand motifs and different isoforms of MLCs may modulate the Ca^2+^ sensitivity of force generation and cross-bridge kinetics (discussed below) [22]. A key difference between MLC2 and MLC1 is a serine residue of the MLC2, which MLC1 lacks, in the N-terminal of the peptide. MLC2 are regulated through Ca^2+^-mediated phosphorylation of this residue, which causes the MLC2 to undergo conformational changes from a compacted to an elongated form [23]. MLC1 on the other hand has a unique N-terminal that binds actin, thus contributing to the cross-bridge cycle kinetics, i.e., force production [24, 25].

## Myosin and the cross-bridge cycle

**Fig. 4:**
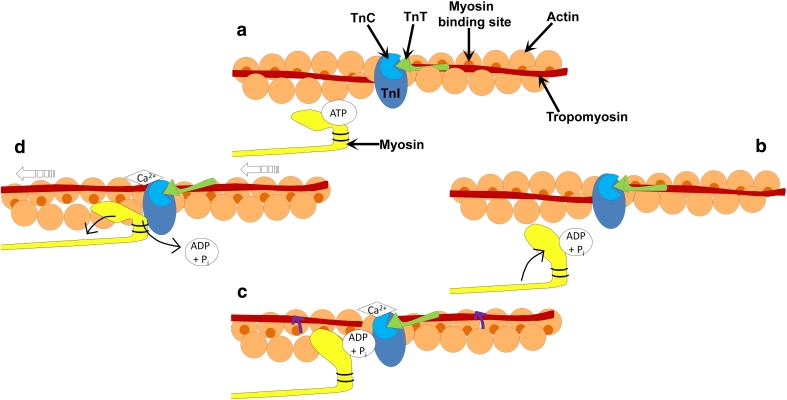
Schematic diagram of the cross-bridge cycle. **a** ATP binds to the ATP-binding domain on the myosin head. **b** ATP is hydrolyzed to ADP and a phosphate allowing the myosin head to move towards the actin filament. **c** Binding of Ca^2+^ to troponin C (TnC) results in a conformational change in the troponin complex, allowing the movement of tropomyosin around the actin filament (as indicated by the *purple arrows*). **d** Release of the hydrolyzed nucleotides results in the extension of the myosin head permitting the sliding of the filaments (*open arrows*). ATP quickly rebinds to the ATP-binding site on the myosin head, allowing dissociation of the myosin away from the actin filament, and the cycle is repeated

Force generation and muscle contraction are produced by the cyclic interactions of the myosin head with actin filaments, which is fuelled by ATP and regulated by Ca^2+^. ATP controls the affinity that myosin has to actin during the cycle. The ATP-binding site on the myosin head binds ATP (Fig. 4a), which in turn hydrolyzes to produce ADP and an inorganic phosphate (Fig. 4b) [26]. This causes myosin to bind to actin (at the actin-binding site) via weak ionic interactions (Fig. 4c). It is at this point that Ca^2+^ regulates the interactions between the myosin head and the actin filament, thus initiating conformational changes in the head region of myosin. Isomerization of the subfragment-1 unit of myosin associated with the release of the inorganic phosphate and strong myosin-actin bonding, results in extension of the 20-kDa lever arm of the myosin molecule, allowing sliding of the two filaments (Fig. 4d) [27]. ADP is then released and ATP quickly rebinds to the nucleotide-binding region [28]. The myosin head therefore dissociates from the actin and the cycle is complete [29, 30].

Ca^2+^ regulates the cross-bridge cycle in striated muscle, via the movement of tropomyosin, allowing hydrophobic interactions to occur between myosin and actin resulting in a tighter bond between the two molecules [31]. Therefore, Ca^2+^ activates the cross-bridge cycle through the thin filament regulatory system. At low levels of Ca^2+^ within the sarcoplasm (cytoplasm of the muscle cell), Ca^2+^ is not bound to the calcium-specific binding sites of troponin C (TnC). TnC is weakly bound to TnI and TnT, but TnI is strongly bound to actin, which holds tropomyosin in a position that blocks the myosin-binding sites on the actin filaments (i.e., the blocked state). When Ca^2+^ is released by the sarcoplasmic reticulum and intracellular concentrations of Ca^2+^ are increased, cross-bridge cycling is turned on. Ca^2+^ binds to TnC inducing a conformational change of the troponin complex [32]. The strength of the interaction between TnC and TnI increases, weakening the interaction between TnT and tropomyosin, TnI and tropomyosin, and TnI and actin. This results in TnI being pulled away from the actin filaments. This causes a 30° shift in the tropomyosin molecule around the thin filament, thus exposing myosin-binding sites that it once covered on the actin filaments (Fig. 4c) [33]. Actin interacts with myosin in a stereospecific manner (the closed state). As Ca^2+^ continuously increases, the transition from a weak to strong cross-bridging pushes the tropomyosin further from its closed position on the actin filament and allows for the complete uncovering of myosin-binding sites and leads to power stroke and generation of force and movement [34]. Figure 3 shows the relationship myosin has to actin, the troponin complex, and tropomyosin.

Phosphorylation of MLC2, via Ca^2+^/calmodulin-dependent myosin light-chain kinases, increases the mobility of myosin cross-bridges such that the myosin heads move away from the thick filament towards actin thin filaments in striated muscle fibers [35]. MLC2 phosphorylation increases the number of cross-bridges entering the contractile cycle by upregulation of the actin-induced phosphate release from the weakly bound actin-myosin ADP-P state. This leads to an increase in Ca^2+^ sensitivity of the myofilament and increases the rate of force development by increasing cross-bridge transition to the strongly bound, force-generating state, while slowing the rate of decay of the force-generating state [36]. In smooth muscle and nonmuscle myosins, Ca^2+^ release is a major determinant of contraction, by activating the Ca^2+^/calmodulin-dependent protein kinases that phosphorylate MLC2 [37]. This phosphorylation increases myosin ATPase activity, resulting in cross-bridge cycling [38]. For detailed reviews on this area, see [39–41].

## The myosin heavy-chain genes

The nomenclature for the myosin II proteins has varied within the literature. For the purposes of this review, the genes have been named according to the nomenclature described by the HUGO gene nomenclature committee (http://www.genenames.org). However, as the myosin proteins are found as hexameric molecules, it was deemed inappropriate to name using the same terminology as the gene. Therefore, the protein has been named using traditional terminology. To avoid confusion, but to also be consistent with the literature, myosin heavy chain is abbreviated to MYH when used to name genes and MHC to name protein product.

Thirteen genes have been described for mammalian MYH including nine sarcomeric muscle genes, three nonmuscle genes, and one smooth muscle gene. Of the sarcomeric muscle genes, six skeletal MYH genes (*MYH1*, *MYH2*, *MYH3*, *MYH4*, *MYH8*, and *MYH13*) are grouped together on human chromosome 17p. The three other striated muscle genes are cardiac MYH genes, *MYH6* and *MYH7* (located on chromosome 14q11.2–q13) and *MYH7B* (chromosome 20q11). There are three nonmuscle myosin II isoforms in humans; *MYH9* (chromosome 22q11.2), *MYH10* (chromosome 17p13) and *MYH14* (chromosome 19q13.33). The smooth muscle gene is *MYH11* (chromosome 16p13.11).

As will be reviewed below, the MYH genes that are expressed to the heart in development are the sarcomeric myosins *MYH3*, *MYH6*, *MYH7*, and *MYH7B* and the nonsarcomeric nonmuscle myosins *MYH9*, *MYH10*, and *MYH14*. The smooth muscle myosin *MYH11* is absent to the heart but present to parts of the vasculature including the aorta. Although these genes are all expressed to the heart or great vessels, clear roles for all in developmental processes within the human cardiovascular system have yet to be fully elucidated. However, model organisms have provided interesting insights into functional roles for many of these genes, which will be discussed below.

The protein product from the *MYH3* gene is named as embryonic myosin heavy chain (eMHC) and *MYH6* is called alpha myosin heavy chain (αMHC) in this review though sometimes as atrial myosin heavy chain (atrial MHC) within the literature. The *MYH7* product is referred to as beta myosin heavy chain (βMHC) in this review though ventricular myosin heavy chain (ventricular MHC) has also been used. Finally, the nonmuscle myosin *MYH9* is referred to as NMHC IIA, *MYH10* is termed NMHC IIB and *MYH14* is called NMHC IIC. The smooth muscle myosin *MYH11* product is usually named smooth muscle myosin heavy chain (SM-MHC*)* within the literature and has been used in this review. For a summary of gene names, human chromosomal location and protein name, see Table 1.

**Table 1:** Chromosomal location and nomenclature of myosin II

Gene name	Human chromosomal location	Commonly used protein name
*MYH3*	17pter–p11	Embryonic myosin heavy chain (eMHC)
*MYH6*	14q11.2–q13	Alpha myosin heavy chain (αMHC)
		Atrial myosin heavy chain (atrial MHC)
*MYH7*	14q11.2–q13	Beta myosin heavy chain (βMHC)
		Ventricular myosin heavy chain (ventricular MHC)
*MYH7B*	20q11	Myosin heavy chain 7B
*MYH9*	22q11.2	Nonmuscle myosin heavy chain IIA (NMHC IIA)
*MYH10*	17p13	Nonmuscle myosin heavy chain IIB (NMHC IIB)
*MYH11*	16p13.11	Smooth muscle myosin heavy chain (SM-MHC)
*MYH14*	19q13.33	Nonmuscle myosin heavy chain IIC (NMHC IIC)
*MYL2*	12q24.11	Myosin light chain 2 ventricular (MLC2v)
		Regulatory light chain ventricular (RLCv)
*MYL3*	3p21.3–21.2	Myosin light chain 1 ventricular (MLC1v)
		Essential light chain ventricular (ELCv)
*MYL4*	17q21.32	Myosin light chain 1 atrial (MLC1a)
		Essential light chain atrial (ELCa)
		Embryonic myosin light chain
*MYL7*	12q13.2	Myosin light chain 2 atrial (MLC2a)
		Regulatory light chain atrial (RLCa)

Only myosin heavy chains and myosin light chains that are expressed in the heart are listed

The gene name is the approved nomenclature according to HUGO (http://www.genenames.org)

## The myosin heavy chains and the cardiovascular system

**Fig. 5:**
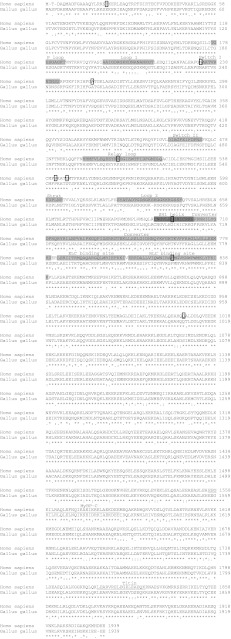
Comparison of human and chick αMHC protein sequences. The human αMHC protein sequence (NP_002462) is compared to the chick sequence (NP_001013415), with various structural domains denoted on the human sequence [158]. The sequences were aligned in ClustalW2 [159, 160]. The nucleotide (ATP)-binding pocket is in part composed of P loop, Loop I, and Switch I with Switch II also important in its function. The rigid relay loop is proposed to connect the ATP binding site to the converter domain. The Strut and Loop 2 are regions that bind the upper and lower 50-kDa subdomains. Switch II is thought to be important in forming a kink, and allowing movement of the converter domain. The converter domain is a socket for the carboxy terminal helical tail and is where rotation occurs around the SH1 helix (also termed the “fulcrum” within the literature), allowing bending of the molecule. The proposed domains for the binding of titin and myosin binding protein-C (MyBP-C) are also denoted (*underlined*) [161, 162]. *ELC* essential light chain, *RLC* regulatory light chain, *asterisk* fully conserved residues, *colon* residues with strongly similar properties conserved;* period* residues with weakly similar properties conserved. Missense mutations previously described in the human MYH6 gene [2, 58, 59] and listed in Table 2 are denoted (*boxed*)

Each myosin heavy-chain gene, which plays a role, or potential role, in heart development is reviewed and the expression and function each gene plays in the heart is described for different animal models. Table 2 summarizes the known effects of altered gene expression during cardiogenesis. αMHC is extensively homologous across species and Fig. 5 illustrate this between the human and the chick, with many important functional domains showing 100 % homology. This degree of conservation across species shows the importance of the MHC proteins.

**Table 2:** Genes encoding myosin structural proteins associated with the developing heart

Gene	Species	Mutation/effect on gene expression	CHDs associated with mutation/developmental process	References
*MYH3*	Chick	Knockdown	Ab atrial and trabeculae development; enlarged heart; abnormal AP and calcium and potassium transients	[42]
*MYH6*	Human	I820N	ASD	[2]
		A230P	TA	[58]
		H252Q	TGA, PFO	
		E501Stop	TA	
		V700M	PFO	
		A1366D	AS, SDK, SAR, PFO	
		A1443D	ASD	
		R1865Q	ASD, DIVC, VSD	
		IVS37-2A > G	ASD, PTA	[59]
		R17H	ASD, AVSD, SVC/CS	
		C539R	ASD	
		K543R	ASD	
		A1004S	ASD	
	Chick	Knockdown	Ab atrial septal development	[2, 61]
			Ab trabeculae development; looping defects, EH	[61]
			Ab calcium transients in atrium	[42]
	Mouse	Homozygous	Death E11–12.5, heart phenotype ND	[60]
		Heterozygote	Viable, fertile, Ab cardiac function, fibrotic lesions, Ab sarcomeres	[60]
	Zebrafish	Weak atrium homozygous	Absent contraction, Ab myofibrillogenesis in atrium	[53]
	Xenopus	Muzak homozygous	Absent myofibrils and cardiac contraction, EH	[62]
*MYH7*	Human	R281T	ASD, EA	[65]
		Y283D	ASD, VSD, pulAH	[66]
		Y350N	EA	
		L390P	EA, PFO	
		K1459N	EA	
		N1918K	Coa/BAV	
		E1573K	VSD	
		1220delE	EA	
	Chick	Knockdown	Ab calcium transients in atrium and ventricle	[42]
			EH	UnD
	Zebrafish	Half-hearted	Enlarged ventricle, fewer myofibrils, increased cardiomyocytes	[70]
	Medaka fish	Hozuki mutant	Enlarged ventricle, increased cardiomyocytes	[71]
*MYH10*	Mouse	Homozygous	VSD, DORV, hypertrophic cardiac myocytes	[78]
*MYH11*	Human	IVS32 + 1G to T	TAAD	[82]
		R1758Q		
		R1241_L1264del		
		L1264P	TAAD, PDA	[83]
		R1275L		
		R712Q		
		R669C	PDA	[84]
		E1290Q		
	Mouse	Homozygous	PDA	[85]
*MYL2*	Mouse	Homozygous null	Death E12.5, EH, wall thinning, Ab sarcomeres	[97]
	Chick	Knockdown	Ab cardiac looping, Ab sarcomeres	[91]
*MYL4*	Zebrafish	Knockdown	Ab sarcomeres with increased length, decreased contractility	[110]
*MYL7*	Mouse	Homozygous null	Death E10.5–11.5, EH tube, Ab looping, Ab trabeculae, left ventricular dilation, Ab myofibril assembly	[121]
	Zebrafish	Knockdown	Ab sarcomeres with decreased length and contractility, EH	[110]
		Tell tale homozygous	Ab contraction, Ab sarcomeres	[120]

Only mutations and phenotypes related to cardiac development are described; mutations and phenotypes related to cardiac function e.g., cardiomyopathy, are not listed

*Ab* abnormal, *AP* action potential, *AS* aortic stenosis, *ASD* atrial septal defect, *AVSD* atrioventricular septal defect, *BAV* bicuspid aortic valve, *Coa* coarctation of the aorta, *DIVC* dilated inferior vena cava, *DORV* double outlet right ventricle, *E* embryonic day, *EA* Epstein’s anomaly, *EH* enlarged heart, *ND* not determined, *PDA* patent ductus arteriosus, *PFO* persistence of foramen ovale, *PTA* persistent truncus arteriosus, *PulAH* pulmonary artery hypoplasia, *SAR* subaortic ridge, *SDK* septal dyskinesis, *TA* tricuspid atresia, *SVC/CS* abnormal drainage of superior vena cava to coronary sinus, *TAAD* thoracic aortic aneurysm and/or aortic dissection, *TGA* transposition of the great arteries, *UnD* unpublished data (Dr. CS Rutland and SL), *VSD* ventricular septal defect

### Myosin heavy chain 3

eMHC is a skeletal myosin heavy chain protein. *MYH3* transcripts are expressed in human fetal skeletal muscle predominately, although they are also expressed by adult skeletal muscle [42]. Roles for *MYH3* have yet to be shown in the human heart, although expression has been seen by in situ hybridization in human 4-, 5.5-, and 7-week fetal hearts, with RNA localized in the myocardium of the atrium, ventricle, and sinus venosus [42]. The chick *MYH3* gene is expressed in the myotome, skeletal muscle, and chick heart from HH12 (an early stage of cardiac looping) through to the adult heart [42–45]. In the chick, eMHC staining was detected in the myocardium of the atrial, ventricular, and outflow regions of the developing heart [42]. In addition, immunoreactivity at HH9 was detected in a heart-specific manner, demonstrating this structural protein is expressed from the earliest stages of cardiogenesis.

Mutations in human *MYH3* have been associated with Freeman-Sheldon and Sheldon-Hall syndromes, both syndromes associated with skeletal defects [46, 47]; defects to the heart were not described. However, upon knockdown of eMHC during early cardiogenesis in the chick, the atrial septa and trabeculae developed abnormally [42]. Further, both atrial and ventricular cardiomyocytes formed an abnormal action potential (AP) and had decreased intracellular K^+^ and Ca^2+^ transient spikes. With regards to the ventricular cells, most were electrically inactive. These data suggest that the structural protein eMHC is a candidate gene for atrial septal defects (ASD; an abnormal opening between the left and right atria chambers) and conduction anomalies [42]. Analysis of *MYH3* has not been performed in any other animal models to our knowledge.

### Myosin heavy chain 6

The expression profile of αMHC protein is similar in the chick and human, both during development and postnatally. Expression is observed in skeletal muscle and the heart during development and in the adult. During cardiac development, although expression is found to be higher in the atria, expression is seen in the ventricles, with the ventricular expression decreasing relative to the atria as development progresses [48–51]. In the adult heart, αMHC is predominately expressed in the atrium with very low levels found in the ventricles [48–50]. In rodents, however, although *Myh6* is predominantly expressed to the atrial region during embryogenesis, a presence is also demonstrated in the ventricle, and *Myh6* becomes the dominant myosin heavy-chain isoform after birth in both the atrial and ventricular chambers [50, 52]. The orthologue to *MYH6* in zebrafish is *atrial MYH*, which is atrial-specific during embryogenesis (zebrafish form a two-chambered heart) [53]. In *Xenopus* (which forms a three-chambered heart with two atria and one ventricle), the orthologue to *MYH6* is also called *atrial MYH*. *Atrial MYH* is the dominant myosin heavy-chain gene during early frog cardiogenesis, with expression throughout the myocardium [54]. Expression is also throughout the heart of the adult frog.

Despite intensive screening, relatively few mutations have been found in the *MYH6* gene that have been linked to cardiomyopathy [55–57], suggesting low penetrance of this phenotype compared to genes such as *MYH7* (see below). With regards to a role in the developing heart, αMHC was the first structural protein that upon mutation of its gene was associated with a CHD, with members of a family carrying a *MYH6* mutation afflicted with an ASD [2]. This mutation caused a hydrophobic isoleucine to change to a hydrophilic asparagine (I820N). This missense mutation (18,429 T > A) was in the neck region and was predicted to affect the binding of the myosin heavy chain to its regulatory light chain. Subsequently, a number of missense mutations, a splice site and a nonsense mutation have been found in *MYH6* [58, 59]. Defects were not exclusive to ASDs, with a number of other CHDs found including other septal anomalies ventricular septal defect (VSD; an abnormal opening between the left and right ventricular chambers) and persistent truncus arteriosus (PTA; the septum between the pulmonary trunk and aorta fails to form). Functional analysis of three of the missense mutations suggests that these mutations affect the normal formation of the myofibrils, with A230P and A1366D disrupting and H252Q enhancing assembly [58]. The location of all these missense mutations are denoted on the αMHC protein in Fig. 5.

A number of animal models have provided a greater understanding of this gene. In the mouse null mutant, loss of *Myh6* led to embryonic lethality, with death occurring at embryonic day (E) 11–12.5 [60]. However, the embryonic heart phenotype was not characterized. In the heterozygote animals, mice were found to be viable and fertile with no overt phenotype. Upon detailed cardiac function studies, however, adult heterozygotes (12–25 weeks post-birth) had defects in cardiac contraction and relaxation with incomplete penetrance, in comparison to wild-types [60]. Further, histological analysis revealed fibrotic lesions in heterozygote mice. In the chick, knockdown of αMHC has demonstrated roles for this structural protein in the initiation and/or maintenance of the atrial septum [2, 61]. Further, development of the ventricular trabeculae and the structural architecture of the atrial septum, was on occasion found to be aberrant [61]. In addition, at a low penetrance, abnormal cardiac looping and an enlarged heart were observed. Individual atrial and ventricular cardiomyocytes from HH19 knockdown hearts had normal AP characteristics, although cytosolic Ca^2+^ appeared to show modest changes in atrial (but not ventricular) cells compared to controls [42]. These data suggest that αMHC may not play a critical role in the conduction system early in development. A loss-of-function mutation of the *myh6* gene in zebrafish, called weak atrium, is due to a frameshift mutation with the loss of thymidine at position 4,024 [53]. Loss of *myh6* in zebrafish caused absent atrial contraction and abnormal myofibrillogenesis, with secondary defects consisting of thickening of myocardial wall and a decrease in ventricular lumen size [53]. Despite these defects, homozygous weak atrium mutants can survive to adulthood and heterozygotes appeared normal [53]. A nonsense mutation (a C to T transition at position 3,187) of *myh6* in *Xenopus tropicalis*, the muzak mutant, resulted in deletion of the coiled-coil domain [62]. These mutants have absent myofibrils and the heart fails to contract, which led to a number of presumed secondary effects including absent trabeculae and cardiac valves. Heterozygotes had no discernible phenotype [62].

### Myosin heavy chain 7

*MYH7* is considered to be the ventricular myosin heavy-chain gene. The βMHC protein is expressed mainly in the ventricle in both the fetal (from 47 days, the earliest stage analyzed) and adult human heart [48, 49]. During embryogenesis in rodents, expression of *Myh7* also becomes ventricular-specific, seen from E7.5 in the mouse [50, 52]. In addition, in contrast to other species, expression of *Myh7* is downregulated postnatally in rodents, so that in the adult heart *Myh6* is the dominant myosin heavy-chain gene expressed in both the atrial and ventricular chambers [50, 52]. Outside the heart, βMHC is also expressed in skeletal muscle during development and in the adult. The expression profile of *MYH7* in the chick is similar to that in humans [48–51]. The presumptive functional orthologue to *MYH7* in *Xenopus* is not expressed in the frog heart prior to chamber formation; subsequently from stage 45 expression is seen in the regions between the ventricle and outflow tract, and between the ventricle and atria [54]. Ventricular- and outflow-specific expression is seen in the adult frog. Ventricular-specific expression of *myh7* is seen during and upon completion of chamber specification in the zebrafish embryo [53, 63].

Although numerous mutations in *MYH7* are known to be associated with cardiomyopathy [64], in recent years CHDs have also been linked to this gene. A large family with left ventricular non-compaction was found to carry a mutation in the cDNA of the *MYH7* gene at 842 G > C, with four individuals also afflicted with an ASD and/or Epstein’s anomaly (EA; malformed tricuspid valve) [65]. This mutation led to a positively charged arginine being replaced by an uncharged threonine (R281T) and was predicted to prevent a salt bridge from forming, leading to instability in the myosin head. A further six missense mutations and one small deletion (of a glutamine residue) have also recently been described, which have been associated with defects including ASD, VSD, and EA [66]. However, the functional significance of these mutations still needs to be elucidated. To our knowledge, transgenic mice with deletion of the *Myh7* gene causing defects during cardiogenesis have not been described. When our laboratory knocked down βMHC during early stages of heart development in the chick, all of the morpholino positive embryos had an enlarged heart, but the atrial septa and other structures within the heart were found to be normal (Dr. Catrin Rutland and SL, unpublished data). Further, as seen with αMHC, the atrial and ventricular cardiomyocytes had normal action potentials, although irregular Ca^2+^ transients were seen in atrial and ventricular cells [42]. Defects in calcium signaling may lead to defects in contraction of the heart, with MHC known to be important for induction of contraction [67–69]. Two fish models have been used to analyze the presumptive functional orthologue to *MYH7*. The zebrafish has a shorter developmental period in comparison to the medaka fish, with hatching occurring at 48 h post-fertilization in the zebrafish and 8 days in the medaka fish. The zebrafish half-hearted mutant forms due to a C to T transition at position 3,094 bp of the cDNA, which results in a stop codon within the tail region and hence a non-functional truncated protein [70]. These mutant embryos have an enlarged and distended ventricular chamber, which fails to contract, with a normally formed atrium. In the medaka fish, the hozuki mutants form due to a nonsense mutation (an A to T transition) in the *myh7* gene that leads to a loss of most of the tail domain [71]. These mutants have an enlarged ventricle, which can be seen from cardiac looping (stage 30), with excessive cardiomyocyte formation and fewer myofibrils in the ventricle. The atrium appears normal and the hozuki mutant embryos survived until hatching.

### Myosin heavy chain 7b

In the adult human heart, *MYH7B* transcripts were detected by RT-PCR [72] with the developing heart not investigated. In mice, in situ hybridization showed that *Myh7b* is expressed in the developing heart throughout the myocardium of the atrial and ventricular regions [72]. Expression is also detected in the adult heart, in somites and skeletal muscle and tissues such as the brain and the smooth muscle layer of large blood vessels [72–74]. In the chick, expression of *MYH7B* is found in the developing heart from early stages and in the day 19 post-hatched heart, throughout the myocardium [72], as seen in the mouse. Intriguingly, this expression in the chick contrasts with a previous study that detected *MYH7B* in the Purkinje fibers of the heart just prior to hatching, and absent to the myocardium [72, 75]. This discrepancy is currently not understood. In *Xenopus*, expression of *myh7b* is found in the developing heart and in the adult [72]. Expression was also observed in somites in both chick and *Xenopus*. However, despite the expression of *MYH7B* is now known in a number of species, its function is currently poorly understood.

### Myosin heavy chain 9

The nonmuscle myosin, NMHC IIA, is expressed in the human heart. However, it is expressed in the smooth muscle, endothelial, and fibroblast cells of the heart, not in cardiomyocytes [76]. Expression was seen at two stages; 19 weeks of development and in the adult. The expression of NMHC IIA in the murine heart is similar to human; smooth muscle, endothelial and fibroblast cells express *Myh9*, but cardiomyocytes do not [76]. During development, *Myh9* transcripts are found in the E9.5 mouse and the HH12 chick heart, stages when the heart is undergoing looping in both species [77]. NMHC IIA is also expressed in a number of other tissues such as lung, liver, and kidney. To date, mutations in this gene have not been associated with abnormalities in the heart.

### Myosin heavy chain 10

The nonmuscle myosin protein NMHC IIB is widely expressed. In the heart, it is expressed to smooth muscle cells, endothelial cells, fibroblasts, and cardiomyocytes [76]. Immunostaining found NMHC IIB to be diffuse throughout the cytoplasm in human fetal cardiomyocytes at 19 weeks of gestation. In the adult human heart, NMHC IIB is restricted to the Z-lines and intercalated discs [76]. A similar expression pattern is seen for the mouse. *Myh10* expression is seen in the early looping heart in both mouse (at E9.5) and chick (at HH12) [77].

To our knowledge, mutations in the *MYH10* gene have not been found in humans. However, *Myh10* ablation in the mouse leads to embryonic lethality by E14.5 in most embryos, with a variety of defects in the heart, including VSDs, double-outlet right ventricle (DORV; the origin of the aorta is abnormally located from the right ventricle) and hypertrophic cardiomyocytes seen at high penetrance [78]. Many null *Myh10* embryos upregulated NMHC IIA, a potential compensatory mechanism for non-heart tissues that express *Myh10*. Of the null mutants that were live born, death occurred on postnatal day 1 due to congestive heart failure [78]. The heterozygous mice were normal. Cardiomyocyte-specific knockout of *Myh10*, using the loxP/Cre recombinase system, lead to mutant mice born with hypertrophic myocytes [79]. These mice also had VSDs (seen at low penetrance as expression levels were reduced only from mid-gestation) although DORV was not observed. Subsequently, cardiomyopathy was seen, with the presentation of the phenotype observed between 6 and 10 months postnatally [79]. In addition, the intercalated discs appeared wider than in controls and some of the cardiomyocytes were multinucleated. The related nonmuscle proteins NMHC IIA and NMHC IIC were not found to be upregulated in these mutant mice. A role for NMHC IIB has been suggested in the spreading of cardiomyocytes, and hence in the regulation of cell size [80]. Other animal models have not been investigated to date.

### Myosin heavy chain 11

The protein product of the *MYH11* gene is SM-MHC, which is a contractile protein of smooth muscle cells. It is expressed in cells derived from smooth muscle lineages; expression is seen from the E10.5 aorta in the mouse, and later in development in peripheral blood vessels, intestine, bladder, and uterus [81]. Interestingly, expression is absent to organs such as the heart, kidney, and brain except to the vasculature.

Mutational analysis of *MYH11* was performed in two kindreds afflicted with thoracic aortic aneurysm and/or aortic dissection (TAAD) with three mutations detected [82]. It was proposed that the location of the mutations may affect the coiled-coil structure of the C-terminal region of this smooth muscle MHC protein, and hence the assembly of myosin thick filaments. A dominant-negative effect of the mutations was proposed [82]. Further, symptomatic individuals with the mutation were found to have a lower aortic compliance and a higher pulse wave velocity, leading to a severe decrease in the elasticity of the aortic wall. Subsequently, three *MYH11* missense mutations have been linked to individuals with TAAD and patent ductus arteriosus (PDA; the ductus arteriosus fails to close postnatally) [83, 84] and rarely to isolated PDA [84]. Amino acid residue changes L1264P and R1275L, due to mutations 3,791 T > C and 3,824 G > T, respectively, were located in the coiled–coiled region whereas residue alteration R712Q, caused by the 2,153 C > T mutation, was located in the ATPase head domain [83]. The R712Q mutation was predicted to destabilize the SH1 helix and hence prevent the motor domain and lever arm from communicating effectively. Mutations to the coiled–coiled domain were predicted to affect protein–protein interactions. Histological analysis was performed on tissue from affected individuals; smooth muscle cells were found to be disorganized and show hyperplasia in the aortic media, leading to lumen narrowing in some vessels [83]. Further missense mutations associated with isolated PDA were R669C (mutation 2,005 C to T in the cDNA) and E1290Q (3,868 G to C in the cDNA) [84]. The R669C mutation was in the globular head of SM-MHC, a region predicted to play a role in actin binding, whereas E1290Q was located in the tail. Other variants were also described in this study, but were also seen in controls. Consistent with the PDA phenotype seen in some affected individuals, *Myh11* knockout mice were also found to have delayed closure of the ductus arteriosus [85].

### Myosin heavy chain 14

As with the other nonmuscle myosins, the protein product of *MYH14* is widely expressed. With regards to the heart, NMHC IIC is expressed in cardiomyocytes of the mouse E13.5 heart, with immunofluorescence restricted in the intercalated discs of the adult heart [86]. Mutations in the human *MYH14* gene have not been associated with defects in heart formation or function. Although expressed in the heart, the *Myh14* mouse knockout appeared normal and survived to adulthood [86].

## The myosin light chains

As with the myosin heavy chains, the nomenclature for the myosin light chains has also varied within the literature. In this review, the genes have been named according to the nomenclature described by the HUGO gene nomenclature committee, but as with the heavy chains, the proteins have been named in line with the general literature. In addition, in this review, myosin light chain is abbreviated to MYL for the gene and MLC for the protein product.

As mentioned above, two types of MLCs exist; the essential (MLC1; also known in the literature as ELC or alkali MLC) and regulatory light chains (MLC2; also known as RLC or phosphorylatable MLC). Both types are associated with the neck region of the MHC. To date, eight genes encode mammalian MLC, with each isoform having a distinct expression profile. There are four MLC1 genes: *MYL1* (chromosome 2q24.11), *MYL3* (chromosome 3p21.3), *MYL4* (chromosome 17q21.32) and *MYL6* (chromosome 12q13.2). *MYL1*, *MYL3*, and *MYL4* are expressed in striated muscle, while *MYL6* is a nonmuscle and smooth muscle myosin. There are also four MLC2s: the sarcomeric *MYL2* (chromosome 12q24.11), *MYL5* (chromosome 4p16.3) and *MYL7* (chromosome 12q13.2), and the smooth muscle *MYL9* (chromosome 20q11.23). For a summary of gene names, human chromosomal location and protein name, see Table 1.

*MYL3*, *MYL4*, *MYL2*, and *MYL7* are expressed in the heart in a restricted manner during development and have been shown to play a key role in cardiogenesis. Again, the nomenclature for the MLCs has varied in the literature. *MYL3* is also known as MLC1v, ELCv or VLC1, while *MYL4* is commonly referred to as the MLC1a, embryonic MYL, ELCa or ALC1. *MYL2* of the MLC2s are also named MLC2v or RLCv, and *MYL7* is known as MLC2a or RLCa.

## The myosin light chains and the cardiovascular system

The expression and function for each myosin light-chain gene, which plays a potential role in heart development, is discussed. The effects of altering MYL gene expression on the developing heart are summarized in Table 2.

### Myosin light chain 2

MLC2v is restricted to the ventricles, both throughout the developing and adult human heart [87]. Expression of *Myl2* is also restricted to the ventricular cardiomyocytes of the heart tube in rodents (mouse and rat) [88, 89]. As the heart tube begins to fold, expression is also detected in the proximal outflow tract; however this expression only remains until ventricular septation commences [89]. Chick expression of *MYL2* appears similar to that of the mouse and always remained restricted to the ventricular portion of the heart, but is also detected as early as HH5, prior to heart tube formation [90, 91].

Mutations of the human *MYL2* gene have been associated with hypertrophic cardiomyopathy [92–97]. Lack of Mlc2v in mice is embryonic lethal (E12.5) due to cardiac dysfunction that results in heart failure. Hearts dissected from these embryos showed massive cardiac enlargement, wall thinning, dilation of the chambers, and pleural effusions. Upon ultrastructural analysis of these hearts, abnormalities in myofibril assembly were seen, displaying disorganized alignment of the thick and thin filaments, narrow fibers, and larger distances between Z-bands in the homozygous null in comparison to wild-types [97]. Knockdown of *MYL2* in the chick resulted in cardiac anomalies, including irregular heart looping and, again, poorly developed sarcomeres such that Z-discs appeared as dense irregular shapes instead of properly formed bands [91].

### Myosin light chain 3

MLC1v expression is restricted to the ventricular segment of the linear heart tube throughout development and in the adult human heart [98]. This expression is also seen in the mouse [99]. In the* Xenopus*, *Myl3* expression is detected in the somatic mesoderm during the tail bud stage and from stage 31, just prior to heart tube formation, and is detected in the precursor cells of the myocardium, but becomes restricted to the ventricular region of the heart after looping [100].

MLC1v is expressed in the atria of children with peri-membranous VSDs and tetralogy of Fallot (a CHD that involves four defects—overriding aorta, pulmonary stenosis, VSD, and right ventricular hypertrophy) [101]. Mutations of *MYL3* have been associated with familial hypertrophic cardiomyopathy (FHC), and, although these mutations are rare when compared with mutations in *MYH7*, the outcomes of these mutations are quite malignant. Ten mutations of the *MYL3* gene have been associated with FHC, all of which have been found on the EF-hand domain of the protein [94, 96, 102–107].

### Myosin light chain 4

Human embryonic whole hearts express MLC1a, as well as in skeletal muscle [108]. However, postnatally, protein levels decrease to undetectable levels in the ventricles but remain in the atria [109]. Mlc1a in *Xenopus* is extensively expressed in myocardial cells at stage 31 [100]. Zebrafish express only one MLC1, cmlc1, in a cardiac-specific manner and this MLC1 is the orthologue to human MLC1a [110].

A change in expression of MLC1a to other regions apart from the atrium postnatally has been associated with CHDs and cardiomyopathies, linked with pressure overload. Children with tetralogy of Fallot were shown to express large amounts of MLC1a in the ventricles, replacing the endogenous MLC1v of this region [111]. This was also the case in the hypertrophied left ventricle of patients with ischemic, dilative, and hypertrophic cardiomyopathy [112–114]. These isoform switches appear to be compensatory mechanisms of the heart, causing increased cycling kinetics of the cross-bridge cycle, and hence, regulating contraction of the affected heart [22]. This isoform switch was also studied in the mouse. Transgenic overexpression of *Myl4* leads to high levels of expression in the ventricles, replacing the endogenous *Myl3* [115]. Although the isoform shift was benign, with no pathology observed, there was improved cardiac function in the mouse hearts. Knockdown of *cmlc1* in zebrafish resulted in failure of the assembly of Z-bands from the Z-bodies [110]. In addition, the thick filaments appear less dense as they fail to align and assemble properly into A-bands within the sarcomere during development, increasing sarcomeric length. End systolic ventricular volume of *cmlc1* knockdowns was greater than that of wild-types indicating the morphant hearts can dilate but not contract sufficiently [110]. These results suggest a vital role for MLC1 in sarcomeric assembly and fine-tuning of cardiac contractility.

### Myosin light chain 7

MLC2a is expressed in humans at high levels in the atrium postnatally, throughout the linear heart during development, and can also be detected in the adult ventricles, but at lower levels [116]. In the mouse, *Myl7* is initially expressed throughout the linear heart tube early in development (E7.5); however, it becomes restricted to the atria after E12.5 [117]. Expression is similar in the rat [88]. Although expression is seen throughout the linear heart tube, the protein is incorporated in the myofibrils of the atria only, not into the ventricular myofibrils [97]. The *mlc2* gene is the *Xenopus* orthologue of human *MYL2* [118]. It is expressed early in development in the cardiac mesoderm and in subsequent steps of heart tube formation, looping, and chamber septation where it is not restricted to any one area of the myocardium [119]. Zebrafish show strong expression of only one isoform of MLC2s in the heart, cmcl2, which is thought to be the zebrafish orthologue of human MLC2a [110, 120]. *Cmcl2* is expressed in the 13 somite stage (prior to heart tube formation) zebrafish embryo, and is expressed throughout the myocardium of the heart by the time heart looping has occurred [63].

Mutations in *MYL7* have not yet been associated with human disease to our knowledge. *Myl7* null mice were found to be embryonic lethal between E10.5–11.5, and unusual cardiac morphogenesis was apparent in the early linear heart tube (E7.5) such as enlargement of the heart tube and abnormal morphogenesis in the looping heart tube. They also presented with enlarged atria and outflow tracts. The ventricles displayed thin walls, with underdeveloped trabeculae and left ventricular dilation was apparent. At the ultrastructural level, the myofibrils in the atria were disorganized, with a lack of alignment of the thick and thin filaments, associated with diminished beating in the atrial chamber, while cardiomyocytes in the ventricles appeared normal [121]. Chimeric mice of chromosome 21, used as an animal model for Down’s syndrome, showed post-transcriptional down-regulation of endogenous *Myl7*, which was also seen in human patients [122]. As more than 50 % of Down’s syndrome patients have a CHD, this study suggests a potential role for MLC2a in CHDs. Knockdown of *cmcl2* using morpholino oligonucleotides in the zebrafish results in a number of defects in cardiogenesis. Assembly of the dotted Z-bodies into Z-discs failed and previously assembled thick filaments did not align into A-bands, with the sarcomeres of decreased length. Cardiac contractility was reduced, as was the ventricular chamber volume [110]. These data indicate that MLC2s are important in myofibrillogenesis and cardiac contractility. Mutations in the *cmcl2* gene were also studied. The tell-tale mutation *tel* ^*m225*^ is a fully penetrant embryonic lethal recessive mutation that perturbs cardiac contractility in early embryonic development [120]. Although heart tube formation appears normal, with the two heart chambers of normal size, strong peristaltic contractions of the chamber seen in the wild-type are weaker in the *tel* ^*m225*^ mutant. This is the result of disturbances of thick filament assembly of the sarcomere, suggesting a role for MLC2 in sarcomerogenesis [120].

## The role of transcription factors in the regulation of myosin

Structural proteins are the downstream targets of transcription factors that control heart formation in a tightly controlled manner. Therefore, considering the critical role these transcription factors play in cardiogenesis, it is not surprising that a number of these genes have also been found to form CHDs when mutated. The first cardiac transcription factor associated with a CHD was in 1998 in the homeobox gene *NKX2.5*, with mutations found in probands predominately afflicted with an ASD and conduction defects [123]. Subsequently, mutations have been found in numerous other cardiac transcription factors, such as *GATA4* [124], and the T-box genes *TBX5* [125, 126] and *TBX20* [127]. It is also of interest that many of these transcription factors have synergistic effects, and are upstream of other cardiac transcription factors or genes such at the natriuretic factors *NPPA* (natriuretic peptide precursor a, also known as atrial natriuretic factor) and *NPPB* (natriuretic peptide precursor b, also known as brain natriuretic factor) [128–132]. Serum response factor (*SRF*) is a ubiquitous transcription factor that elicits its effect on cardiac and smooth muscle genes by associating with its cofactor myocardin. Myocardin is also known to interact with a number of other cofactors [133].

As stated above, βMHC is specifically expressed to the ventricle [48, 49]. In zebrafish, the lack of *myh7* expression in the atrium is regulated by Nkx2.5 [134]. The zebrafish homeobox transcription factor Prx2 and the mouse Gata factors Gata4 and Gata6 have also been shown to regulate *myh7* expression [131, 134, 135]. However, in embryonic hearts isolated from compound heterozygote *Gata4/Tbx5* mice, mRNA expression of *Myh7* was found to be unaffected, unlike *Myh6*, which showed decreased expression in the compound heterozygote but not in the single *Gata4* and *Tbx5* heterozygotes [130]. *Myh6* is also regulated by important cardiac transcription factors. Both GATA4 and TBX5 activate *Myh6* expression in rodents in vitro [2, 132, 136]. In addition, the transcription factor myocardin, which is a cofactor for SRF and TBX5, activated *Myh6* [137, 138]. In contrast, *Gata4*/*Gata6* compound heterozygote mice did not show decreased *Myh6* expression, despite these mice having a range of heart defects [131]. Bmp-4 is also thought to be important in the mediation of sarcomeric myosin expression in *Xenopus* [139]. IRX4 (iroquois homeobox 4) is a transcription factor that is ventricular specific; it has a role in myosin regulation by activating *MYH7* to be expressed in the ventricle while suppressing *MYH6* [140]. Activation of the *Myh7b* mouse promoter was shown to occur via Gata, Mef2, and E-box binding sites, with the Mef2 site being the most important [72]. Interestingly, other conserved regulatory elements were found to be important in *Myh7b* expression, although the proteins that bind to these sites have yet to be identified.

In comparison to the sarcomeric myosins, the regulation of the nonsarcomeric myosins in the heart is poorly understood. GATA6 has been shown to be important in the activation of the smooth muscle *Myh11* gene, with GATA6 forming a complex with the transcriptional coactivator p300 [141]. In contrast to *Myh6*, *Myh11* was not activated by the myocardin/TBX5 complex [142]. However, myocardin has been shown to activate smooth muscle *Myh11* via other cofactors including SRF [142–144].

MYLs are also regulated by a number of transcription factors. Unlike mammals, *Xenopus* has only one regulatory MYL. Enhancer elements within the promoter region of this gene have been shown to be essential for the heart-specific expression in *Xenopus* [119]. Additional in vitro studies in *Xenopus* of the Gata motifs and an SRF site within this MYL promoter region were shown to be necessary for this specific expression, and over-expression of *gata6* has been associated with a lack of mlc2 expression [119, 145]. Further, *gata* and *srf* genes were shown to act synergistically in regulation of the regulatory *myl* in the frog [119]. This is supported by another study in *Xenopus* that demonstrated that regulatory *myl* is activated via the srf cofactor myocardin interacting with gata4; this contrasted with *myh6* which could be activated by each factor independently [138]. As stated previously in this review, MLC phosphorylation is regulated by myosin light chain kinase (MLCK). Interestingly, Mlck is also regulated by the cardiac transcription factor Nkx2.5 [146].

## Micro RNAs in the regulation of myosin

Additional regulation of cardiac transcription factors and myosin genes is via noncoding RNAs, notably miRNAs. miRNAs are known to be important in a number of biological processes, such as cell proliferation, differentiation, and apoptosis [147]. miRNAs modulate gene expression predominately by acting as negative regulators by inducing the degradation or inhibiting the translation of target mRNAs. Mature miRNAs are approximately 22 nucleotides long, with their formation involving two RNase III enzymes Drosha and Dicer [148, 149]. The extent to which miRNAs play in heart development is currently not fully understood, though is likely to be complex, as any one miRNA may regulate more than one target, and more than one miRNA can bind to the same gene. The role that miRNAs play in cardiac transcription factor regulation is beyond the scope of this review, with a number of excellent reviews recently published [147, 150]. However, it is relevant to summarize the recent data demonstrating a role for miRNAs in the regulation of the myosin genes.

Intergenic, intronic, and exonic miRNAs can occur, with the exonic miRNAs being expressed with the host gene. Intergenic and intronic miRNAs have their own regulatory elements. Embryonic stem cells undergo mesodermal differentiation to a myocardial lineage, with miR-1 and miR-133 playing critical roles in controlling this process [151, 152]. Further, miR-1 has been shown to be important for cardiomyocyte differentiation and its overexpression was found to repress *Myh6* [152, 153]. With regards to the myosin genes having their own miRNAs, three intronic miRNAs have been located within cardiac myosin heavy-chain genes. miR-208a is located in intron 27 of the human and mouse *MYH6* gene [154], with the related miR-208b located in intron 31 of *MYH7* [73] and miR-499 is within intron 19 of the *MYH7B* gene [155]. In the mouse, the expression of these miRNAs correlates with their host genes, with miR-208a mainly expressed in the adult heart, miR-208b predominately expressed in the developing heart and miR-499 expressed during cardiac morphogenesis and in the adult [74, 156]. Although miR-208a, miR-208b, nor miR-499 seem to function during mouse cardiogenesis, miR-208a was shown to be the dominant miRNA in the adult heart, by upregulating *Myh7* transcripts in response to stress [73, 154]. However, as other larger animals express MYH6 and MYH7 differently to mice, the expression and function of miR-208a and miR-208b could also be different. Consistent with this, an in vitro study using human cardiomyocytes progenitors suggests a role for miR-499 in differentiation [153], with overexpression of miR-499 in human embryoid bodies leading to significant upregulation of *MYH7* [155].

## Conclusions and future perspectives

Myosin structural proteins are expressed in a restricted manner in the developing heart and play vital roles in early cardiogenesis. In this review, we have shown that changes in expression can lead to detrimental effects on the developing heart, with a variety of phenotypes seen. However, there remain many gaps in knowledge with as yet a comprehensive understanding of the role myosin heavy and light chains play in cardiogenesis still lacking. In the future, further genetic and molecular studies will elucidate precise roles for the myosins in heart development and how the myosins are regulated at different developmental stages. Such insights should improve genetic counseling and may lead to therapeutic treatments of CHDs, cardiomyopathies, and other cardiac anomalies. Already myosin-activating drugs have gone on trial for the treatment of heart failure. These activators work by increasing the transition rate of the weakly bound acto-myosin complex to a strongly bound one, therefore increasing cardiac contraction [157]. A greater understanding of the role and regulation of myosin structural proteins should help pave the way for creating a detailed molecular map of heart development.
